# COLLEGE COMPLETION AS A PROTECTIVE FACTOR FOR LATER-LIFE COGNITION: ISSUES OF SELECTION

**DOI:** 10.1093/geroni/igz038.219

**Published:** 2019-11-08

**Authors:** Emily Greenfield, Ayse Akincigil, Sara Moorman

**Affiliations:** 1 Rutgers University, New Brunswick, New Jersey, United States; 2 Rutgers, The State University of New Jersey, New Brunswick, New Jersey, United States; 3 Boston College, Chestnut Hill, New Jersey, United States

## Abstract

Additional years of education is considered a modifiable protective factor against Alzheimer’s disease and related dementias. However, some empirical studies have suggested that linkages between educational attainment and later life cognition are largely a function of differential selection into higher education. Our study uses data from the Wisconsin Longitudinal Study, as one of the longest-running cohort studies in the U.S., to further probe how differential selection into higher education might influence associations between college completion and later life cognition. Using adjusted inverse probability weighting and with particular attention to adolescent IQ, we find evidence that college completion is associated with better language for both men and women at age 65, as well as with better memory for men. Examining heterogeneous treatment effects, we further find that associations between college completion and later life cognition are strongest for men who were least likely as adolescents to attend college.

